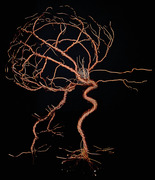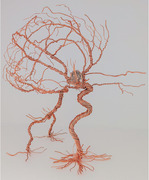# Reflections in Coiling

**DOI:** 10.1161/SVIN.121.000236

**Published:** 2022-01-12

**Authors:** Samantha Spellicy

**Affiliations:** ^1^ Medical College of Georgia Augusta GA

Artist: Samantha Spellicy is an 8th‐year MD‐PhD student currently applying to neurosurgery residency. She completed her PhD in stroke research and is interested in vascular neurosurgery.

About the art: Multigauge copper wire sculpture reflecting 3D relationships of cerebrovascular anatomy

Artist perspective: I had the opportunity to rotate on a vascular neurosurgery elective during my 4th year of medical school, an invaluable opportunity for a neurosurgery residency applicant. From my first angiogram, I was struck by the absolute beauty of the cerebrovasculature system in all its seemingly nonsensical array. Although I peered at 2D angiograms striving to detect aneurysms shadowed by familiar arteries and vessels, I worked to reconstruct the 3D relationships in my head. I wanted to bring that picture in my head to fruition. I knew I had to make a model.

After watching the vascular neurosurgeon tirelessly, skillfully, and elegantly coil aneurysms with wires of different rigidity, diameters, and lengths, I knew wire was the perfect medium. I had not made a wire sculpture since high school, but quickly the technique came back. I found the dexterous process of creation almost as satisfying as the end product and immediately recognized the educational and meditative value. Through this process, I gained a better understanding of the 3D angles, vessel‐to‐vessel relationships, shadows cast, and volumetric footprints of a “coiled” aneurysm.

What struck me the most after finishing the piece was that it felt alive and almost reminiscent of a creature moving, reaching out, and walking across a plane. It seems art imitates life.